# Gut microbiota from androgen‐altered donors alter pulmonary responses to ozone in female mice

**DOI:** 10.14814/phy2.14584

**Published:** 2020-10-14

**Authors:** Ross S. Osgood, Hiroki Tashiro, David I. Kasahara, Vladimir Yeliseyev, Lynn Bry, Stephanie A. Shore

**Affiliations:** ^1^ Department of Environmental Health Harvard T.H. Chan School of Public Health Boston MA USA; ^2^ Massachusetts Host‐Microbiome Center Department of Pathology, Brigham & Women’s Hospital Boston MA USA

**Keywords:** airway hyperresponsiveness, androgens, G‐CSF, IL‐17A, microbiome

## Abstract

In mice, both androgens and the gut microbiota modify pulmonary responses to ozone. We hypothesized that androgens affect gut microbiota and thereby impact pulmonary responses to ozone. To address this hypothesis, we transferred cecal microbiota from male castrated or sham castrated C57BL/6J mice into female germ‐free recipient C57BL/6J mice. Four weeks later mice were exposed to ozone (2 ppm) or room air for 3 hr. The gut microbiomes of castrated versus sham castrated donors differed, as did those of recipients of microbiota from castrated versus sham castrated donors. In recipients, ozone‐induced airway hyperresponsiveness was not affected by donor castration status. However, compared to mice receiving microbiota from sham castrated donors, mice receiving microbiota from castrated donors had elevated numbers of bronchoalveolar lavage (BAL) neutrophils despite evidence of reduced lung injury as measured by BAL protein. Serum concentrations of IL‐17A and G‐CSF were significantly greater in recipients of castrated versus sham castrated microbiota. Furthermore, BAL neutrophils correlated with both serum IL‐17A and serum G‐CSF. Our data indicate that androgen‐mediated effects on the gut microbiota modulate pulmonary inflammatory responses to ozone and suggest a role for circulating IL‐17A and G‐CSF in these events.

## Graphical abstract

While sex differences in ozone‐induced airway hyperresponsiveness (AHR) are apparent in mouse models, the role of androgens in ozone‐induced AHR have not been examined. Our data confirm previous reports that gut microbiota have the potential to impact host inflammatory responses to ozone and indicate that effects of these microbiota on systemic inflammation may contribute to responses to ozone. A better understanding of the mechanisms by which sex hormones manipulate the gut microbiome may ultimately lead to improved treatment of lung diseases such as asthma.

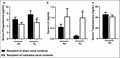

## 1 INTRODUCTION

Ozone (O_3_) is a trigger for asthma. Both emergency room visits and hospital admissions for asthma increase on days with high ambient O_3_ concentrations (Fauroux, Sampil, Quenel, & Lemoullec, 2000). Inhaled O_3_ causes airway epithelial damage, release of acute‐phase cytokines and chemokines, and recruitment of neutrophils and macrophages into the lungs (Devlin et al., 1991; Holtzman et al., 1979; Kasahara et al., 2015; Michaudel et al., 2018; Mumby, Chung, & Adcock, 2019). O_3_ also causes symptoms of asthma (Goodman et al., 2018), declines in lung function (Yang et al., 2005), and airway hyperresponsiveness (AHR), a defining feature of asthma (Alexis et al., 2000; Bell, Dominici, & Samet, 2005; Holtzman et al., 1979). Interestingly, asthmatics differ in their susceptibility to O_3_ (Vagaggini et al., 2010). Understanding the factors that contribute to this susceptibility may ultimately prove important for the prevention or treatment of O_3_‐induced lung dysfunction.

Sex is one of these factors. There are sex differences in the pulmonary response to O_3_. Compared to young adult women, young adult men have diminished lung function following long term O_3_ exposure (Galizia & Kinney, 1999) sure to O_3_ is associated with emergency room visits in males of all ages, whereas in females the association is only evident after the age of 10 (Sheffield, Zhou, Shmool, & Clougherty, 2015). We and others have established that there are also sex differences in pulmonary responses to O_3_ in mice: O_3_‐induced AHR is greater in male than female mice (Birukova et al., 2019; Brown, Tashiro, Kasahara, Cho, & Shore, 2020; Cho, Abu‐Ali, et al., 2019; Kasahara et al., 2019).

Sex hormones likely contribute to sex differences in O_3_‐induced AHR (Birukova et al., 2019; Fox, Adams, Brookes, & Lasley, 1993; Fuentes, Cabello, Nicoleau, Chroneos, & Silveyra, 2019; Osgood, Kasahara, Tashiro, Cho, & Shore, 2019). In mice, responses to O_3_ are greater when exposure occurs during the follicular stage of the estrous cycle when estradiol and luteinizing hormone (LH) are elevated than during the luteal stage when these hormones decline but progesterone rises (Fox et al., 1993; Fuentes et al., 2019). Compared to sham castrated controls, castrated male mice are protected against O_3_‐induced AHR and pulmonary inflammation (Osgood et al., 2019). In male mice, the administration of the androgen receptor antagonist, flutamide, also attenuates O_3_‐induced AHR and pulmonary inflammation (Osgood et al., 2019). The data indicate that both male and female hormones have the capacity to impact pulmonary responses to O_3_, but the mechanistic basis for the effects of these hormones remains to be established.

The gut microbiome affects pulmonary responses to O_3_. In lean male mice, both antibiotics and germ‐free (GF) conditions attenuate the AHR and neutrophil infiltration induced by acute O_3_ exposure (Cho et al., 2018). Interestingly, these effects are driven by gut rather than lung microbiota. Vancomycin, an antibiotic that specifically targets Gram‐positive species and is not absorbed from the gut (Vinolo, Rodrigues, Nachbar, & Curi, 2011), significantly reduces inflammatory responses to O_3_ (Cho et al., 2018).

Sex differences in the gut microbiome have been reported in both mice and humans (Bolnick et al., 2014; Cho, Abu‐Ali, et al., 2019; Dominianni et al., 2015; Haro et al., 2016; Org et al., 2016). In both mice and humans, sex hormones can also shape the composition of the gut microbiome (Org et al., 2016; Yatsunenko et al., 2012). In turn, changes in the gut microbiome also alter sex hormones (Markle et al., 2013). Finally, sex differences in gut microbiota occur after but not before puberty (Markle et al., 2013; Steegenga et al., 2014) and gut microbiota of castrated male mice and pigs more closely resemble females than males (He et al., 2019; Yurkovetskiy et al., 2013). These sex differences can have functional consequences for sex‐biased diseases. In non‐obese diabetic mice, a model for type 1 diabetes (T1D), females have almost twice the rate of T1D as males. However, this sex difference is abolished in mice lacking a microbiome (Markle et al., 2013). Moreover, protection against T1D can be transferred to a female mouse through a transfer of gut microbiota from a male donor (Markle et al., 2013). Sex differences in O_3_‐induced AHR are also abolished by antibiotic treatment, suggesting that the microbiome contributes to these sex‐biased responses (Cho, Abu‐Ali, et al., 2019). Consistent with these data, female mice exposed to male mouse microbiota through housing in cages with dirty bedding from male mice develop responses to O_3_ similar in magnitude to those of male mice (Cho, Abu‐Ali, et al., 2019).

While we have established that androgens augment the pulmonary response to O_3_ (Osgood et al., 2019), the mechanistic basis for the role of androgens remains to be established. The purpose of this study was to determine how androgen‐altered microbiota might modify pulmonary responses to O_3_. Analyses leveraged conventional cecal donor microbiota from castrated or sham castrated male mice that were gavaged into female GF mice. Four weeks following gavage, responses to O_3_ were assessed in the recipient females. We used female rather than male GF recipients to reduce the impact of the recipients’ own androgens on the gut microbiome as it established in recipients.

## 2 METHODS

### 2.1 Mice

This study was approved by the Harvard Medical Area Standing Committee on Animals. For cecal transfer studies, donor male C57BL/6J mice were purchased from The Jackson Laboratories. Removal of the gonads by castration was performed at 4 weeks of age. Controls underwent sham castration surgery. Unlike humans, mouse adrenal glands do not produce peripheral androgens (van Weerden, Bierings, van Steenbrugge, de Jong, & Schroder, 1992). Thus, androgen‐induced influences on gut microbiota in donor castrated mice should be low. Each of the donors, whether castrated or sham castrated was housed in the same room at the Jackson Laboratories. No antibiotics were used during recovery from surgery. These donor mice were transferred to our vivarium at 5 weeks of age and housed until 10 weeks of age at which time cecal contents were harvested. Briefly, whole cecum was removed and transferred to large cryovials for flash freezing in liquid nitrogen until use. Frozen materials were thawed in a Coy anaerobic chamber in 50 ml conical tubes with 5 ml of sterile, pre‐reduced PBS supplemented with 0.05% cysteine and homogenized. Female C57BL/6J recipient GF mice were obtained from the gnotobiotic breeding colonies maintained at the Massachusetts Host Microbiome Center. Recipient mice were gavaged with cecal contents from the male donors at 10 weeks of age. All mice were housed under a 12/12 hr light/dark cycle, fed regular mouse chow, and housed with at least two mice per cage.

**FIGURE 1:**
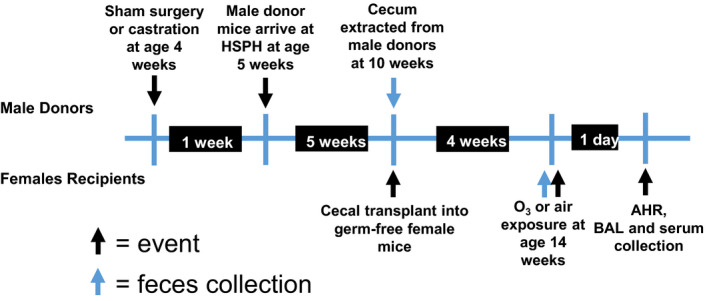
Protocol for cecal transfer experiment

Our experimental protocol is outlined in Figure 1. Two‐hundred μl of the cecal content slurry from castrated or sham castrated male C57BL/6J 10‐week‐old donor mice was gavaged into age‐matched GF C57BL/6J female mice. An aliquot from each donor was saved for 16S rRNA sequencing. Female recipient mice were housed in gnotobiotic isolators at the Massachusetts Host‐Microbiome Center until exposed to air or O_3_ 4 weeks later. Four weeks were allowed between gavage and evaluation to ensure the maturation of the recipients’ microbiota and associated gut, immunologic, and other physiologic responses (Fransen et al., 2017). Four weeks after cecal transfer, fecal pellets were harvested from the recipient mice for 16S rRNA gene sequencing to determine microbiome composition. Mice were then exposed to room air or O_3_ (2 ppm for 3 hr). Exposure began between 9 and 10 o'clock a.m. each day. Twenty‐four hours following exposure, mice were anesthetized for the measurement of airway responsiveness to inhaled aerosolized methacholine. Following the measurement of airway responsiveness, mice were euthanized with an overdose of sodium pentobarbital, blood was harvested by cardiac puncture for the preparation of serum, and bronchoalveolar lavage (BAL) was performed.

Experiments were replicated with five cohorts of recipient mice. Each cohort consisted of eight mice—four received the cecal contents of a single castrated donor mouse and four received the cecal contents of a single sham castrated donor mouse. Half of each group was exposed to air and half to O_3_.

### 2.2 Ozone exposure

During exposure to O_3_ (2 ppm, 3 hr) or room air, female recipient mice were placed in individual wire mesh cages within a stainless steel and Plexiglas chamber. Food and water were removed during exposure and mice were returned to clean cages with food and water immediately following exposure (Lu et al., 2006). In mice, exposure to an O_3_ concentration of 2 ppm for 3 hr results in an O_3_ dose that is equivalent to that used in human chamber studies in which subjects are exposed to a 0.4 ppm O_3_ for 2 hr with intermittent exercise (Hatch et al., 1994; Watkinson, Highfill, Slade, & Hatch, 1996). This latter exposure regimen produces AHR in human subjects (Seltzer et al., 1986).

### 2.3 Measurement of pulmonary mechanics and airway responsiveness

Following exposure to room air or O_3_, mice were anesthetized with pentobarbital and xylazine, intubated with a tubing adaptor, and ventilated (flexiVent, SCIREQ) as previously described (Cho, Abu‐Ali, et al., 2019; Cho et al., 2018). A common volume history was established by three volume excursions to total lung capacity (30 cm H_2_O trans‐respiratory system pressure). Next, measurements of total respiratory system resistance (*R*_RS_) were obtained after the administration of an aerosol of PBS and after the administration of increasing concentrations of aerosolized methacholine chloride (1–100 mg/ml). After each measurement of (*R*_RS_), total lung impedance (*Z_L_*) was measured and partitioned into components representing Newtonian resistance (Rn), reflecting the conducting airways, and the coefficients of respiratory system tissue damping (*G*) and respiratory system tissue elastance (*H*), reflecting the lung tissue and peripheral airways. At each dose, the three highest values of *R*_RS,_ Rn, *G*, and *H* measured in the 3 min period after aerosol administration were averaged to construct dose‐response curves.

### 2.4 BAL and blood collection

Following measurement of airway responsiveness, blood was collected from the right ventricle via cardiac puncture with a 12‐gauge needle for the preparation of serum. To isolate serum, blood was placed in BD Microtainer Blood Collection Tubes (#365967) and allowed to clot for at least 30 min before the centrifugation and isolation of serum per kit instructions. Mice were then euthanized and the lungs were lavaged with two 1 ml of aliquots of PBS. BAL fluid was combined and centrifuged to separate supernatant and cells. Total BAL cell counts were counted with a hemacytometer and cell differentials were determined by centrifuging BAL cells onto glass slides and staining with a Hema3 Stain kit. At least 300 cells were counted. The BAL supernatant was frozen at −80°C until analyzed for protein content by the Thermo Scientific Pierce BCA protein assay kit. The BAL supernatant was concentrated 8‐fold (Amicon Ultra—0.5 ml centrifugal filters, Millipore) before cytokines and chemokines were assayed by the multiplex assay (Mouse Cytokine Array/Chemokine Array 31‐Plex [MD31], Eve Technologies). Reported values are those present in the original unconcentrated samples. BAL IL‐17A was measured by enzyme‐linked immunosorbent assay (ELISA) following the manufacturer's instructions (BioLegend, #43250). Serum was prepared and stored at −80°C until the analysis of cytokines and chemokines by the multiplex assay (Eve Technologies).

Analysis of serum sex hormones by ELISA was conducted at the University of Virginia Ligand Core. Commercial kits were used for the measurement of progesterone (IBL America, #IB79183), estradiol (Calbiotech, #ES180S‐100), and testosterone (IBL America, #IB79174). Mouse LH was measured using an in house method at the University of Virginia Ligand Core with an assay sensitivity of 0.04 ng/ml. (https://med.virginia.edu/research‐in‐reproduction/contact‐us/ligand‐assay‐analysis‐core/assay‐methods). Due to limited serum, testosterone was only measured in air‐exposed mice.

### 2.5 16S rRNA sequencing

Fecal pellets were collected from female recipients of sham or castrated cecal contents immediately before O_3_ or room air exposure. All fecal pellets were stored at −80°C until DNA was isolated. The All Prep Power Viral DNA/RNA Kit (Qiagen) was used to isolate fecal DNA. 16S rRNA gene sequencing over the V4 region by MiSeq (Illumina) was performed by the Massachusetts Host‐Microbiome Center at Brigham and Women's Hospital as described (Bucci et al., 2016). Microbiome data analysis was performed using the Nephele pipeline. This pipeline includes analysis with Qiime software using the Greengenes 99 database (Caporaso et al., 2010; Weber et al., 2018). In all cases, differential abundance and association with experimental variables were assessed using Multivariate Association with Linear Models (MaAsLin) statistical framework (Morgan et al., 2012). A q‐value of ≤0.25 (*p*‐value corrected using the Benjamini‐Hochberg correction method) and *p*‐value of <.05 was considered significant.

### 2.6 Statistics

Except for 16S rRNA sequencing analysis (see above), significant differences between groups were assessed using Factorial ANOVA combined with LSD Fisher post hoc analysis (Statistica Software) using fecal donor status (sham castrated or castration) and exposure (air or O_3_) as the main effects, except where only two groups were compared (e.g., BAL cytokines, body weight). A *p* < .05 (two‐tailed) was considered significant. Values are expressed as mean ± *SE*.

### 2.7 Data and software availability

16S rRNA raw sequencing files (fastq) were uploaded to the NIH Sequence Read Archive (SRA) (entry PRJNA558992). The Nephele pipeline used for 16S sequencing analysis can be found at https://nephele.niaid.nih.gov. MaAsLin analysis was conducted using galaxy at http://huttenhower.org/galaxy.

## 3 RESULTS

### 3.1 Effect of cecal transfer on the gut microbiome

**FIGURE 2:**
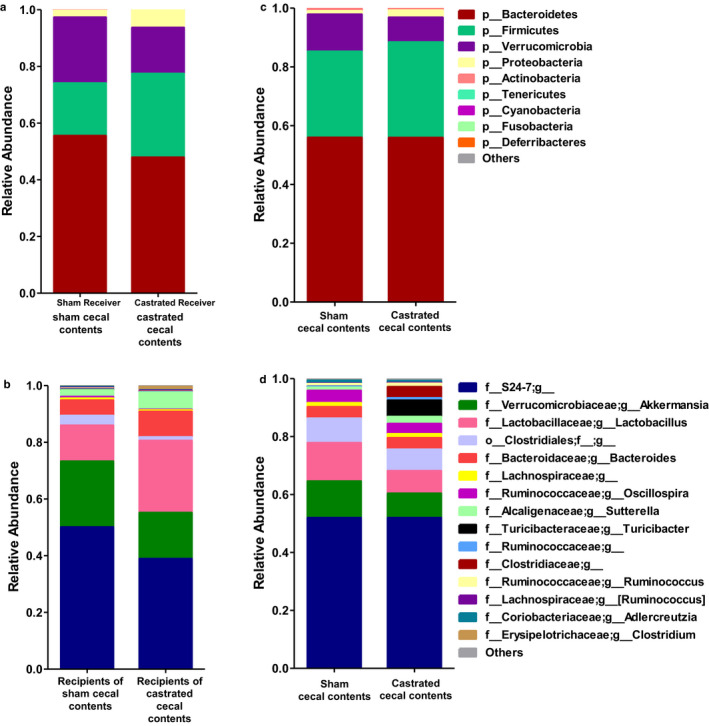
Impact of cecal transfer on the gut microbiome. 16S rRNA sequencing was performed on DNA prepared from cecal contents of donor mice and fecal pellets of recipient mice. For the latter, fecal pellets were harvested before exposure. Donors were not exposed. (a) relative abundance of bacterial phyla from fecal pellets from recipients of cecal contents from sham castrated versus castrated donors; (b) relative abundance of top 15 genus‐level taxa in fecal pellets of recipients of sham castrated and castrated cecal contents; (c) relative abundance of bacterial phyla in cecal contents from sham castrated and castrated donors; (d) relative abundance of top 15 genus‐level taxa from cecal contents of sham and castrated cecal contents; *n* = 20 mice per group for recipient mice and *n* = 5 mice per group for donor mice. Samples for 16S rRNA sequencing were taken from one cohort of donor mice and five cohorts of recipient mice

To examine the impact of androgen‐exposed microbiota on O_3_‐induced AHR and pulmonary inflammation, we gavaged GF female mice with cecal contents from sham castrated or castrated male mice as detailed in Figure 1. 16S rRNA gene phylotyping analyses of taxon abundance at both the phylum and genus level indicated differences between recipients of the cecal contents of the sham castrated versus the castrated donors (Figure 2a,b). At the phylum level, Actinobacteria and Bacteroidetes were lower and Firmicutes and Proteobacteria were higher in the recipients of castrated versus sham castrated donors (Figure 2a; Table 1). At lower taxonomic classification, *Adlercreutzia* was the dominant signature of Actinobacteria, *S24–7* dominated Bacteroidetes, *Lactobacillus* dominated Firmicutes, and *Sutterella* dominated Proteobacteria (Figure 2b; Table 1). Other Firmicutes that were significantly affected by castration status but that constituted <1% of taxon abundance are listed in Table 1. Statistical analysis with MaAsLin confirmed the statistical significance of these changes and identified several less abundant taxa that were also significantly different in recipients of sham castrated versus castrated cecal contents (Table 1). These data indicate distinct differences in the gut microbiomes of the two groups of recipient mice.

**TABLE 1:**
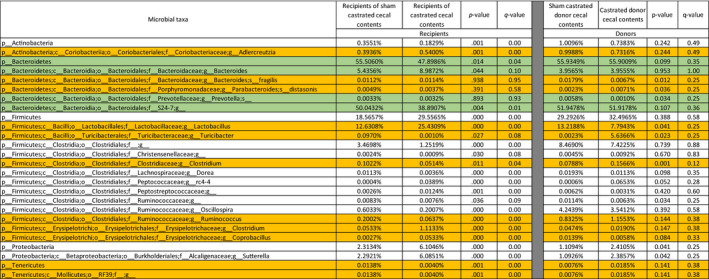
Comparison of the gut microbiomes of donors and recipients.

List of taxa identified as significantly different by MaAsLin analysis (Morgan et al., 2012) between recipients of sham castrated versus castrated cecal contents or between sham castrated versus and castrated donor cecal contents. Comparison of the microbiomes of the donor and recipient mice identified several taxa that were affected similarly in the recipients as in the donors (Taxa highlighted in white). There were also taxa that were affected by castration in one group but not the other (highlighted in green). We also identified taxa that were affected by castration in one direction in the recipients and in the opposite direction in the donors (highlighted in orange). Recipients of sham and castrated cecal contents *n* = 20. Sham and castrated cecal contents *n* = 5.

To determine to what extent the cecal transplant reproduced the gut microbiomes of the donors, we also performed 16S rRNA sequencing on cecal contents harvested from the donors that were used for the transplants. As with the recipients, there were differences in the taxonomic composition of the cecal contents of the sham castrated versus castrated donors (Figure 2c,d and Table 1), consistent with reports of others (Org et al., 2016). At the phylum level, the abundance of Proteobacteria was higher in the cecal contents of the castrated versus sham castrated donors (Figure 2c). *Sutterella* dominated Proteobacteria in the cecal contents of the castrated versus sham castrated donors (Table 1). While the abundance of Firmicutes was not significantly changed, at lower taxonomic classification, the abundance of *Lactobacillus* was lower and the abundances of *Turicibacter* and *Clostridiaceae* were higher in the cecal contents from castrated versus sham castrated donors (Figure 2d). MaAsLin analysis confirmed the significance of these differences and identified other less abundant taxa that were also significantly different in the two groups of donors (Table 1). Taken together, the comparison of the microbiomes of the donor and recipient mice identified several taxa that were affected similarly in the recipients as in the donors (Taxa highlighted in white in Table 1). These included *Sutterella*. However, there were also taxa that were affected by castration in one group but not the other (highlighted in green in Table 1). For example, S24–7 was significantly lower in recipients of castrated than sham castrated cecal contents, but was not different between donor cecal contents (Table 1). There were also taxa that were affected by castration in one direction in the recipients and in the opposite direction in the donors. These included *Lactobacillus*, *Turicibacter*, and *Clostridiaceae* (highlighted in orange in Table 1).

**FIGURE 3:**
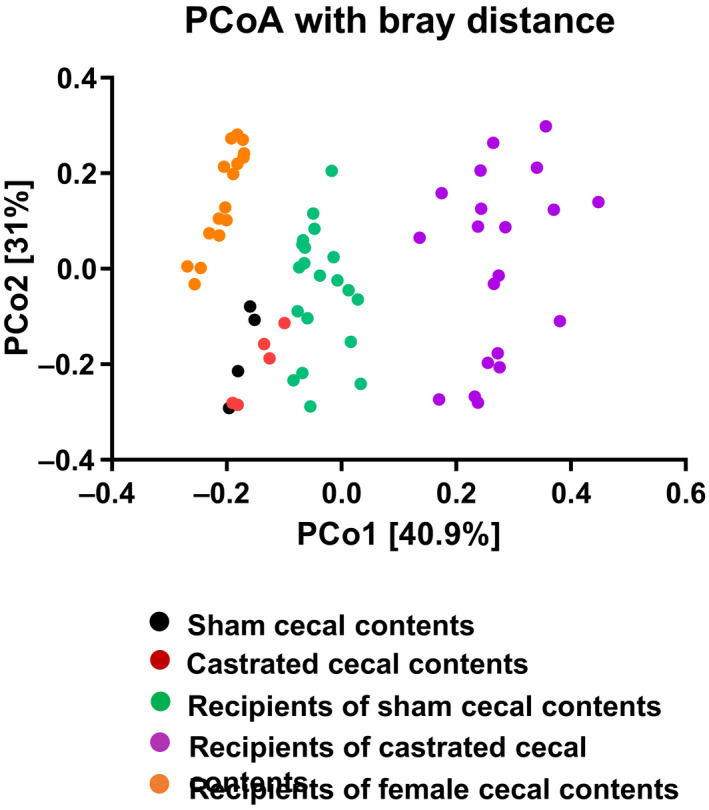
Principal coordinate analysis. 16S rRNA sequencing was performed on DNA prepared from cecal contents of donor mice and fecal pellets of recipient mice. Principal coordinate analysis was calculated by the Bray‐Curtis method; *n* = 20 mice per group for recipient mice; *n* = 5 mice per group for donor mice; *n* = 16 for the female to female recipient mice. Samples were obtained from five cohorts of recipient mice. For comparison, we also included our data from previously published female mice that had been transplanted with cecal contents of female mice (Tashiro et al., 2019). Samples were obtained from four cohorts of recipient mice

Principal coordinate analyses of microbiome datasets from the two groups of male donors and the two groups of female recipients indicated separate grouping of both donors and their respective recipient mice (Figure 3). Thus, while there were some similarities (Table 1), the gut microbiomes of the female recipients of sham castrated or castrated cecal contents were not identical to the gut microbiomes of their sham castrated or castrated donors. Similar results have been reported by others with respect to the transfer of a male microbiome into female mice (Fransen et al., 2017; Markle et al., 2013). For comparison, we also included our data from previously published female mice that had been transplanted with cecal contents of female mice (Tashiro et al., 2019). These mice were grouped separately from either the donor or recipient groups. Notably, the female mice transplanted with cecal contents of female mice (orange circles in Figure 3) grouped closer to the female mice transplanted with cecal contents of male sham castrated mice (green circles) than to those transplanted with cecal contents of castrated male mice (purple circles), indicating greater similarity of their microbiomes. Because the female mice transplanted with cecal contents of female mice were not administered O_3_ at the same time as the other mice in this study, they are not included in the analysis below.

### 3.2 Effect of cecal transfer on pulmonary responses to O_3_

#### 3.2.1 Effect of cecal transfer on O_3_‐induced AHR

**FIGURE 4:**
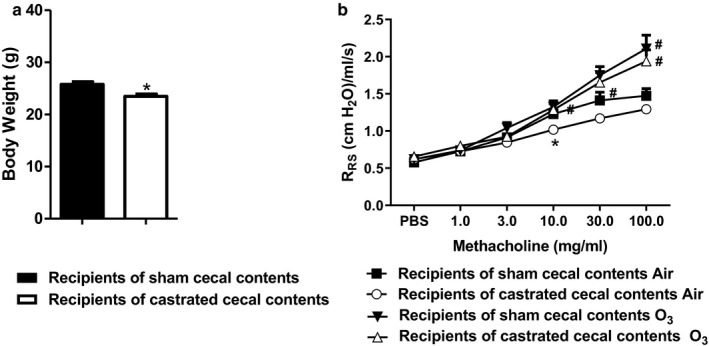
Effect of cecal transfer on O_3_‐induced AHR. Recipients of sham castrated or castrated cecal contents were exposed to room air or O_3_ (2 ppm, 3 hr) and evaluated 24 hr later. Shown are: (a) body weight prior to exposure to room air or O_3_; (b) airway responsiveness of recipients of sham or castrated cecal contents. Results are mean ± *SE* of data from 10 mice per group (2 per group in each of five cohorts). *p < .05 compared with recipients of sham castrated cecal contents with the same exposure; ^#^ *p* < .05 compared to air‐exposed with the same type of cecal content transfer. *R*_RS_, respiratory system resistance

**FIGURE 5:**
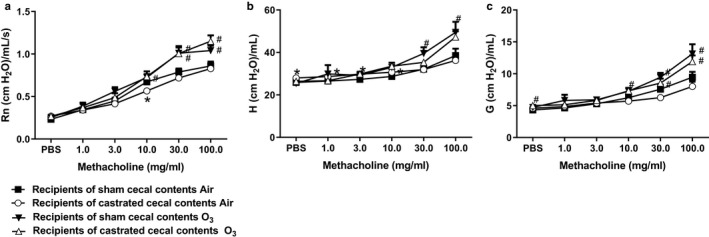
Effect of cecal transfer on methacholine‐induced changes in Rn, *G*, and *H*. Recipients of sham castrated or castrated cecal contents were exposed to room air or O_3_ (2 ppm, 3 hr) and evaluated 24 hr later. Shown are changes in airway responsiveness assessed using (a) Rn, (b) *H*, and (c) *G*. Results are mean ± *SE* of data from 10 mice per group (2 per group in each of five cohorts). *p < .05 compared with recipients of sham castrated cecal contents with the same exposure; ^#^ *p* < .05 compared to air‐exposed with the same type of cecal content transfer. *G*, coefficient of respiratory system tissue damping; *H*, coefficient of respiratory system elastance; Rn, Newtonian resistance

Four weeks after gavage with the cecal contents from sham castrated or castrated mice, female mice were exposed to room air or O_3_. Female mice gavaged with cecal contents from sham castrated mice weighed significantly more than mice gavaged with cecal contents from castrated mice (Figure 4a). In air‐exposed mice, airway responsiveness assessed using *R*_RS_ was lower in the mice that received cecal contents from castrated versus sham castrated mice, an effect that reached statistical significance at the 10 mg/ml of dose of methacholine and near significance (.05 < *p* < .06) at the 30 mg/ml of dose of methacholine (Figure 4b). While O_3_ exposure increased airway responsiveness in both groups of recipient mice, there was no significant difference in airway responsiveness of O_3_‐exposed recipients of sham versus castrated cecal contents (Figure 4b). Similar results were obtained using Rn, *G*, or *H* as the outcome index (Figure 5).

#### 3.2.2 Effect of cecal transfer on serum sex hormones

**FIGURE 6:**
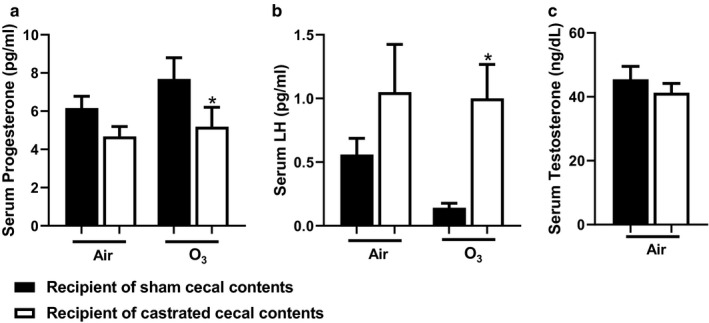
Effect of cecal transfer on host reproductive hormone status. Recipients of sham castrated or castrated cecal contents were exposed to room air or O_3_ (2 ppm, 3 hr). After 24 hr, blood was collected, serum isolated, and sex hormones measured. Shown are serum sex hormones (a) progesterone (b) luteinizing hormone, and (c) testosterone. Results are mean ± *SE* of data from 7 to 10 mice per group from five cohorts of mice. *p < .05 compared with recipients of sham castrated cecal contents with the same exposure

Fecal gavage of male feces into conventional female mice increases serum testosterone (Markle et al., 2013). To test the effect of the cecal transfer on sex hormone concentrations, we measured serum concentrations of estradiol, progesterone, LH, and testosterone. Serum estradiol was measured following both air and O_3_ exposure, but concentrations were below the limit of detection in most samples similar to our previously published data (Cho, Abu‐Ali, et al., 2019). After O_3_ but not air exposure, mice gavaged with cecal contents from castrated mice had significantly less serum progesterone and more LH than mice gavaged with cecal contents from sham castrated mice (Figure 6a,b). In air‐exposed mice, testosterone was unchanged (Figure 6c). Due to limited serum, testosterone was only measured in air‐exposed mice.

#### 3.2.3 Effect of cecal transfer on O_3_‐induced lung inflammation and injury

**FIGURE 7:**
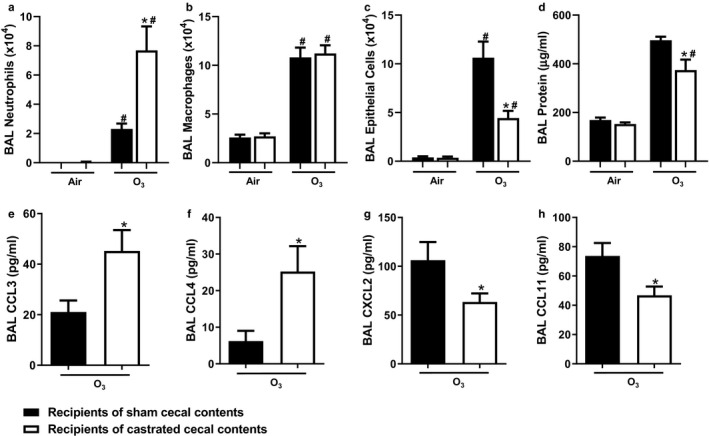
Effect of cecal transfer on O_3_‐induced injury and inflammation. Recipients of sham castrated or castrated cecal contents were exposed to room air or O_3_ (2 ppm, 3 hr) and evaluated 24 hr later. Shown are: (a) BAL neutrophils; (b) BAL macrophages; (c) BAL epithelial cells; (d) BAL protein, (e) BAL CCL3; (f) BAL CCL4; (g) BAL CXCL2; (h) BAL CCL11. Chemokines were assayed with a multiplex assay. Only cytokines and chemokines for which there was a significant effect of castrated versus sham castrated cecal content are shown in this figure. Multiplex was performed only on BAL fluid from O_3_‐exposed mice. Results are mean ± *SE* of data from 9 to 10 mice per treatment group from five cohorts of recipient mice. *p < .05 compared with recipients of sham castrated cecal contents with the same exposure; ^#^ *p* < .05 compared to air‐exposed with the same type of cecal content transfer. For both neutrophil and macrophage cell counts, one statistical outlier was removed from the O_3_‐exposed recipients of castrated cecal contents group that had extremely high neutrophil counts

**FIGURE 8:**
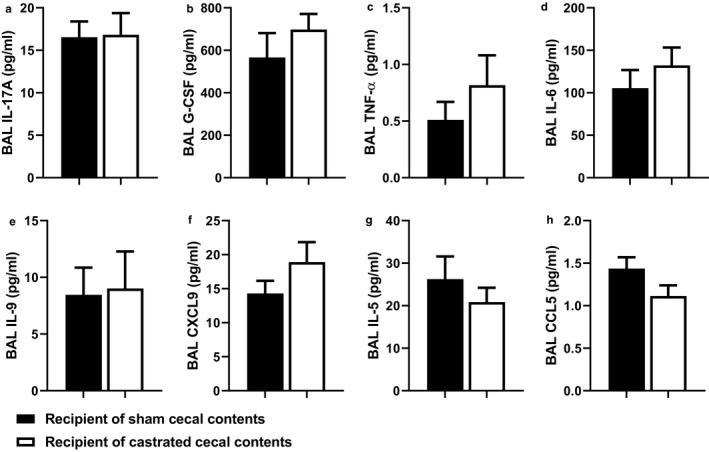
Pulmonary chemokines and cytokines not affected by cecal transplant. Recipients of sham castrated or castrated cecal contents were exposed to O3 (2ppm, 3h) and evaluated 24 h later. Chemokines were assayed with a multiplex assay except for BAL IL‐17A which was measured by enzyme‐linked immunosorbent assay (ELISA). Multiplex and IL‐17A ELISA was performed only on BAL fluid from O3‐exposed mice. Shown are: (a) BAL IL‐17A; (b) BAL G‐CSF; (c) BAL TN Fa‐; (d) BAL IL‐6; (e) BAL IL‐9; (f) BAL CXCL9; (g) BAL IL‐5; (h) BAL CCL5. Results are mea±n SE of data from 9‐10 mice per treatment group from 5 cohorts of recipient mice. There was no significant effect of donor castration status on any of these cytokines and chemokines

Compared to air‐exposed mice, exposure to O_3_ increased BAL neutrophils and macrophages in both groups of recipient mice (Figure 7a,b). While there was no effect of donor status on BAL macrophages in O_3_‐exposed mice, BAL neutrophils were greater in mice receiving castrated than the sham castrated donor microbiota. O_3_ exposure increased BAL epithelial cells in both groups of mice (Figure 7c), consistent with O_3_‐induced damage to airway epithelial cells and subsequent sloughing of these cells. O_3_ also increased BAL protein in both groups of mice (Figure 7d). O_3_‐induced increases in BAL protein reflect damage to the alveolar/capillary barrier and subsequent leak of plasma protein into the lungs (Bhalla, 1999). However, compared to recipients of cecal contents from sham castrated mice, recipients of cecal contents from castrated mice had fewer epithelial cells and less protein in BAL fluid (Figure 7c,d), indicating reduced lung injury. Compared to O_3_‐exposed mice that were recipients of cecal contents from sham castrated mice, recipients of cecal contents from castrated mice had greater levels of BAL CCL3 (Figure 7e) and CCL4 (Figure 7f) but reduced CXCL2 (Figure 7g) and CCL11 (Figure 7h). Both CCL3 and CCL4 have neutrophil chemotactic activity (Bless et al., 2000; Shanley, Schmal, Friedl, Jones, & Ward, 1995), suggesting that increases in neutrophil recruitment observed in the recipients of cecal contents from the castrated mice might the result of effects on CCL3 and CCL4. Other BAL cytokines and chemokines including IL‐17A, G‐CSF, TNFα, IL‐6, IL‐9, CCXL9, IL‐5, and CCL5 were not affected by cecal transplant (Figure 8).

### 3.3 Effect of cecal transfer on systemic inflammation

**FIGURE 9:**
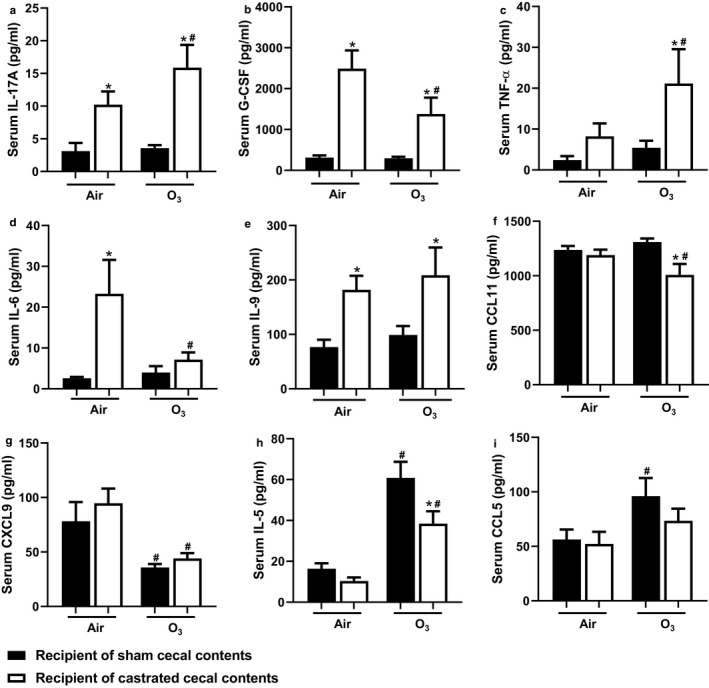
Effect of cecal transfer on O_3_‐induced systemic inflammation. Multiplex assay was performed on serum from both air and O_3_ exposed mice. Shown are: (a) IL‐17A; (b) G‐CSF; (c) TNF‐α; (d) IL‐6, (e) IL‐9; (f) CCL11; (g) CXCL9; and (h) IL‐5, and (i) CCL5. Only cytokines and chemokines for which there was a significant effect of castrated versus sham castrated cecal contents or an effect of O_3_ versus air are shown. Results are mean ± *SE* of data from 5 to 10 mice per treatment group and were obtained from five cohorts of mice. *p < .05 compared with recipients of sham castrated cecal contents with the same exposure; ^#^ *p* < .05 compared to air‐exposed with the same type of cecal content transfer

Certain gut microbiota induce intestinal inflammation, causing an increase in gut permeability and subsequent leak of bacterial products such as endotoxin into the circulation (Cani et al., 2008). This leak can induce systemic inflammation and impact other health outcomes (Cani et al., 2008). Fransen et al (Fransen et al., 2017) reported that compared to the fecal transplant of GF female mice with normal female feces, fecal transplant with normal male feces results in gut inflammation. Consequently, we considered the possibility that there were differences in systemic inflammation in mice that were transplanted with sham versus castrated cecal contents and that these differences might contribute to the augmented neutrophil recruitment observed in mice that were recipients of the castrated cecal contents (Figure 7a). Factorial ANOVA indicated the effects of castration status on certain serum cytokines and chemokines. Compared to recipients of sham cecal contents, recipients of castrated cecal contents had greater serum IL‐17A (Figure 9a), G‐CSF (Figure 9b), TNFα (Figure 9c), IL‐6 (Figure 9d), and IL‐9 (Figure 9e) either after air exposure, after O_3_ exposure, or after either exposure. There were also effects of O_3_ exposure on serum cytokines. Serum G‐CSF (Figure 9b), IL‐6 (Figure 9d), and CCL11 (Figure 9f) were reduced by O_3_ in recipients of castrated cecal contents and serum CXCL9 (Figure 9g) was reduced by O_3_ exposure in both the castrated and sham castrated groups. In contrast, serum IL‐17A (Figure 9a), TNF‐α (Figure 9c), IL‐5 (Figure 9h), and CCL5 (Figure 9i) were increased following O_3_ exposure either in recipients of castrated cecal contents, in recipients of sham cecal contents, or in both groups.

**FIGURE 10:**
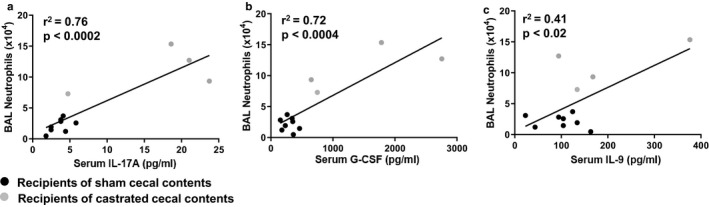
Correlation between BAL neutrophils or cytokines and serum cytokines. Recipients of sham castrated or castrated cecal contents were exposed to O_3_ (2 ppm, 3 hr) and evaluated 24 hr later. Shown are correlations between BAL neutrophils and (a) serum IL‐17A; (b) serum G‐CSF; and (c) serum IL‐9. Note that corresponding data for neutrophils and serum cytokines were only available for four or eight mice per group

It is conceivable that these microbiota‐induced changes in serum cytokines and chemokines contribute to differences in BAL neutrophils observed in recipients of sham versus castrated cecal contents (Figure 7a). Indeed, in O_3_‐exposed mice we observed a significant correlation between serum IL‐17A and BAL neutrophils (*r* ^2^ = .76, *p* < .0002) (Figure 10a) and between serum G‐CSF and BAL neutrophils (*r* ^2^ = .72, *p* < .0004) (Figure 10b). There was also a significant albeit less robust correlation between serum IL‐9 and BAL neutrophils (*r* ^2^ = .41, *p* < .02) (Figure 10c). Neither serum TNFα nor serum IL‐6 correlated with BAL neutrophils.

### 3.4 Androgen‐mediated effects on the gut microbiota correlate with pulmonary inflammatory responses to O_3_

**FIGURE 11:**
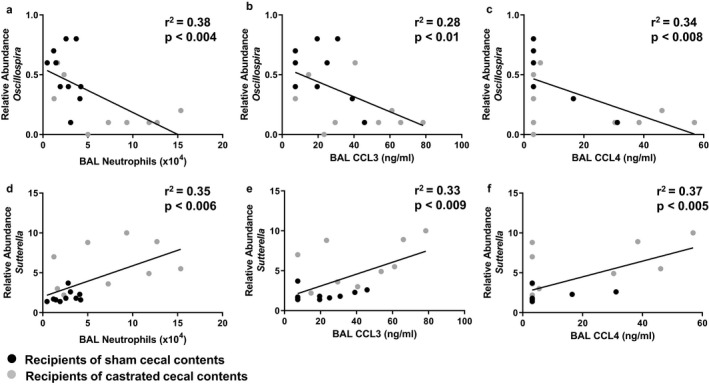
Correlations between gut microbiota and pulmonary inflammation. Recipients of sham castrated or castrated cecal contents were exposed to O_3_ (2 ppm, 3 hr) and evaluated 24 hr later. Shown are correlations between the relative abundances of *Oscillospira* (a–c) and *Sutterella* (d–f) and BAL neutrophils (a, d), BAL CCL3 (b, e), and BAL CCL4 (c, f). Samples were obtained from five cohorts of O_3_‐exposed recipient mice

As described above, there were differences in the gut microbiomes of mice transplanted with cecal contents of castrated versus sham castrated mice (Figures 2 and 3) as well as differences in the subsequent responses of these mice to O_3_ (Figure 7). To determine which affected microbiota might be contributing to observed differences in BAL neutrophils and cytokines (Figure 7), we correlated the relative abundances of taxa identified as significantly different in recipients of sham castrated versus castrated donors with BAL neutrophils, BAL CCL3, and BAL CCL4 measured in the O_3_ exposed mice. We observed significant inverse correlations between *Oscillospira* and BAL neutrophils (*r* ^2^ = .38, *p* < .004) (Figure 11a), BAL CCL3 (*r* ^2^ = .28, *p* < .01) (Figure 11b), and BAL CCL4 (*r* ^2^ = .34, *p* < .008) (Figure 11c). *Oscillospira* abundance is associated with overall health (Konikoff & Gophna, 2016) and is found in lower abundance in patients with inflammatory syndromes (Walters, Xu, & Knight, 2014). We also observed significant correlations between *Sutterella* and BAL neutrophils (*r* ^2^ = .35, *p* < .006) (Figure 11d), BAL CCL3 (*r* ^2^ = .33, *p* < .009) (Figure 11e), and BAL CCL4 (*r* ^2^ = .37, *p* < .005) (Figure 11f). Greater intestinal *Sutterella* abundance is observed with children with food allergies (Zimmermann, Messina, Mohn, Finlay, & Curtis, 2019).

## 4 DISCUSSION

Compared to female mice, males have increased O_3_‐induced AHR (Birukova et al., 2019; Brown et al., 2020; Cho, Abu‐Ali, et al., 2019; Kasahara et al., 2019). In male mice, both castration and flutamide treatment attenuate O_3_‐induced AHR (Osgood et al., 2019). Others have demonstrated that the transfer of male donor microbiota to a female mouse can also transfer sex‐associated phenotypes (Markle et al., 2013). Thus, the goal of this study was to test the hypothesis that androgens modify pulmonary responses to O_3_ by altering the gut microbiota. To do so, we transferred cecal contents from either sham castrated or castrated male mice to naïve conventional GF female mice. However, transfer of the cecal contents of sham castrated versus castrated male donors into GF females did not alter O_3_‐induced AHR, although it did affect innate airway responsiveness (Figure 4b). Surprisingly, we also observed greater increases in BAL neutrophils in the mice that received the cecal contents of castrated versus sham castrated donors (Figure 7a), even though castration per se reduces BAL neutrophils in O_3_‐exposed male mice (Osgood et al., 2019). The changes in neutrophil recruitment (Figure 7a) correlated with the effects of the transplanted microbiota on systemic inflammation (Figures 9 and 10). The data confirm the ability of gut microbiota to alter O_3_‐induced inflammation in the lung and suggest that androgen‐altered gut microbiome may be necessary, but is not sufficient to alter O_3_‐induced AHR.

Given androgen‐dependent effects on gut microbiota (Org et al., 2016) and our own data showing the effects of androgens and of gut microbiota on pulmonary responses to O_3_ (Cho et al., 2018; Cho, Osgood, Bell, Karoly, & Shore, 2019; Osgood et al., 2019), we hypothesized that androgens augment pulmonary responses to O_3_ by altering the gut microbiota. To test this hypothesis, we transferred the cecal contents from castrated or sham castrated male mice into GF female mice. After 4 weeks of colonization, the composition of the resulting microbiota in recipients showed significant differences between the two groups that mimicked some of the differences observed between the two groups of donors (Figures 2 and 3; Table 1). However, the gut microbiota of recipients of sham castrated or castrated cecal contents were not identical to the microbiomes of the cecums of the sham castrated and castrated donors perhaps because of effects of the female host immune response on the male transplanted microbiota as described by others (Fransen et al., 2017). As reported by other investigators, these findings indicate the capacity for both the donor and the recipient to shape the host's microbiota after fecal microbiota transfer (Fransen et al., 2017).

There were greater increases in BAL neutrophils (Figure 7a) in the mice that received the cecal contents of the castrated versus sham castrated donors. We also observed greater BAL CCL3 and CCL4 in the mice that received the cecal contents of the castrated versus sham castrated donors. (Figure 7e,f). We were surprised by these observations since BAL neutrophils and many BAL cytokines and chemokines are reduced rather than elevated in castrated versus sham castrated male mice exposed to O_3_ (Osgood et al., 2019). It is unlikely that the elevations in BAL neutrophils, BAL CCL3, and CCL4 reflect greater lung injury in the recipients of the castrated cecal contents. BAL protein was lower not higher in mice that received cecal contents of the castrated versus sham castrated donors (Figure 7d), though the histological examination of the lung tissue would be needed to confirm these observations. Instead, elevations in BAL neutrophils may result from the effects of the gut microbiome on systemic inflammation and/or on the hormonal status of the recipient mice, as discussed below.

Compared to recipients of sham castrated donors, there were greater systemic inflammation recipients of castrated donors as evident by greater circulating concentrations of a variety of pro‐inflammatory cytokines and chemokines (Figure 9). Moreover, there were strong and significant correlations between serum IL‐17A or G‐CSF and BAL neutrophils (Figure 10a,b). Differences in the serum concentrations of these cytokines are unlikely to be the result of differences in their generation within the lung since neither BAL IL‐7A nor BAL G‐CSF was affected by donor castration status (Figure 8a,b). Instead, there is substantial evidence that certain gut microbiota drives IL‐17A expression within the intestines (Ivanov, 2009; Sugimura et al., 2019; Zhang et al., 2019). Moreover, IL‐17A promotes the expression of G‐CSF which increases the production of neutrophils within the bone marrow (Eyles et al., 2008; Stark et al., 2005). Unfortunately, we did not assess circulating neutrophils in these mice. However, it is plausible that increases in BAL neutrophils observed in O_3_‐exposed mice that received cecal contents from the castrated mice are the result of an IL‐17A/G‐CSF dependent increased pool of circulating neutrophils in these mice.

We do not know exactly what accounts for the increases in systemic concentrations of certain cytokines and chemokines observed in the female recipients of the castrated donors (Figure 9). By 16S rRNA sequencing, recipient and donor mice were free of certain taxa that are known to cause inflammation, such as Helicobacter. However, other microbiota could affect gut barrier function, immune maturation, or priming of host immune responses that ultimately modulated serum IL‐17A and G‐CSF. Even normal male donor microbiota promote intestinal inflammation within female recipients (Fransen et al., 2017), so it is possible that the microbiomes of the recipients of the castrated donors, which were more different from the microbiomes of females than microbiomes of normal male donors (Figure 3), might lead to even greater intestinal permeability and systemic inflammation. Greater intestinal inflammation (Fransen et al., 2017) may also explain the lower body weight in the recipients of the castrated versus sham castrated cecal contents before O_3_ exposure (Figure 4a).

It is also possible that the effects of the gut microbiome on the hormonal status of the recipient mice contributed to differences in BAL neutrophils observed in recipients of castrated versus sham castrated cecal contents. For example, serum LH was greater in the O_3_‐exposed recipients of the castrated versus sham castrated donors (Figure 6b). Others have reported that exposing mice in the follicular stage of the estrous cycle, when LH is high, results in greater O_3_‐induced increases in BAL neutrophils and many BAL chemokines than exposing mice when they are in the luteal stage when LH is low (Fuentes et al., 2019). O_3_ can impact hypothalamic‐pituitary function (Henriquez et al., 2019). We have reported that the gut microbiome also alters O_3_‐induced changes in serum corticosterone and thyroid hormone likely as a result of changes in the release of ACTH and TSH from the anterior pituitary (Cho, Osgood, et al., 2019). The concept that the gut microbiome can impact reproductive hormone status has support in the literature (Kamimura et al., 2019; Markle et al., 2013; Poutahidis et al., 2014). In addition, gut microbiota can impact levels of androgens within the intestinal lumen (Collden et al., 2019). We did not assess androgen levels within the intestinal contents, but serum testosterone was not significantly different between air‐exposed recipients of the castrated versus sham cecal contents.

We also observed reduced airway responsiveness in air‐exposed recipients of donor microbiota from castrated versus sham castrated mice. Similarly, Card et al. (2006) reported a reduction in airway responsiveness in unexposed castrated versus non‐castrated mice. Taken together with the observed castration‐dependent changes in the gut microbiota (Figure 2), the data suggest that the innate airway responsiveness of male mice may be regulated by androgen‐dependent changes in the gut microbiota, changes that were apparent in the absence of O_3_ exposure. Exactly how microbiota affect the airways remains to be established, but microbial metabolites can stimulate sensory nerves in the gut that connect to the brain via the vagus nerve (Bonaz, Bazin, & Pellissier, 2018) and unexposed male mice that have undergone bilateral vagotomy have the reduced airway responsiveness of female mice or castrated mice (Card et al., 2007), suggesting that the autonomic nervous system may be involved.

Whereas airway responsiveness of air‐exposed mice was reduced by cecal donor status, we observed no difference in airway responsiveness in O_3_‐exposed recipients of cecal contents from castrated versus sham castrated mice. The data indicate that androgen‐altered gut microbiome may be necessary, but is not sufficient to alter O_3_‐induced AHR. Nevertheless, we cannot rule out the possibility that the incomplete reproduction of the androgen‐altered microbiota in the female recipients (Figures 2 and 3 and Table 1) accounted for the lack of effect on O_3_‐induced AHR.

Our data confirm previous reports that gut microbiota have the potential to impact host inflammatory responses to O_3_ and indicate that effects of these microbiota on systemic inflammation may contribute to responses to O_3_. A better understanding of the mechanisms by which sex hormones manipulate the gut microbiome may ultimately lead to improved treatment of lung diseases such as asthma.

## CONFLICT OF INTEREST

The authors have no conflicts of interest to disclose.

## AUTHOR CONTRIBUTIONS

R.S.O., H.T., D.I.K., Y.V., L.B., and S.A.S. conceived and designed the experiments. R.S.O. performed the experiments and analyzed the data. V.Y. and L.B. performed and interpreted the 16S rRNA sequencing. R.S.O wrote the paper. R.S.O., H.T., D.I.K., Y.V., L.B., and S.A.S. reviewed, revised, and approved the final version of the manuscript.

## ETHICAL STATEMENT

This study was approved by the Harvard Medical Area Standing Committee on Animals.
